# In-situ experiment reveals CO_2_ enriched fluid migration in faulted caprock

**DOI:** 10.1038/s41598-023-43231-6

**Published:** 2023-10-09

**Authors:** Ulrich Wolfgang Weber, Antonio Pio Rinaldi, Clément Roques, Quinn C. Wenning, Stefano M. Bernasconi, Matthias S. Brennwald, Madalina Jaggi, Christophe Nussbaum, Senecio Schefer, Marco Mazzotti, Stefan Wiemer, Domenico Giardini, Alba Zappone, Rolf Kipfer

**Affiliations:** 1 Department of Geosciences, University of Oslo, Oslo, Norway; 2 Swiss Seismological Service, ETH Zürich, Zürich, Switzerland; 3 Department of Earth Sciences, ETH Zürich, Zürich, Switzerland; 4 Centre for Hydrogeology and Geothermics, University of Neuchâtel, Neuchâtel, Switzerland; 5 Swiss Federal Institute of Aquatic Science and Technology (Eawag), Dübendorf, Switzerland; 6 Swiss Geological Survey, swisstopo, Wabern, Switzerland; 7 Institute of Energy and Process Engineering, ETH Zürich, Zürich, Switzerland; 8 Department of Environmental Systems Science, ETH Zürich, Zürich, Switzerland

**Keywords:** Environmental sciences, Solid Earth sciences

## Abstract

The sealing characteristics of the geological formation located above a CO_2_ storage reservoir, the so-called caprock, are essential to ensure efficient geological carbon storage. If CO_2_ were to leak through the caprock, temporal changes in fluid geochemistry can reveal fundamental information on migration mechanisms and induced fluid–rock interactions. Here, we present the results from a unique in-situ injection experiment, where CO_2_-enriched fluid was continuously injected in a faulted caprock analogue. Our results show that the CO_2_ migration follows complex pathways within the fault structure. The joint analysis of noble gases, ion concentrations and carbon isotopes allow us to quantify mixing between injected CO_2_-enriched fluid and resident formation water and to describe the temporal evolution of water–rock interaction processes. The results presented here are a crucial complement to the geophysical monitoring at the fracture scale highlighting a unique migration of CO_2_ in fault zones.

## Introduction

The injection of CO_2_ for storage into deep geological formations, known as geological carbon storage (GCS), is a climate change mitigation action designed to constrain global warming^1^. For reliable containment, hence long-term storage, the presence of a sealing geological formation located above the storage formation, i.e. the so-called caprock, is required^2^. Clay-rich formations are usually considered caprock analogues because of their low permeability, they act as a barrier to upward migration of the injected CO_2_ arising from buoyancy^3^.

The presence of faults and fractures in a caprock could constitute preferential pathways for free- or dissolved-phase CO_2_ fluid migration^3–5^. Hence, understanding the processes driving CO_2_ mobility within a faulted caprock is important to the design of sustainable management policies for storage operations^3^. Furthermore, quantifying coupled CO_2_ migration and induced fluid–rock interactions under such in-situ heterogeneities is a major challenge that needs to be addressed to guide the assessment and management of storage sites^4^.

The fundamental interactions of CO_2_ with caprocks have been inferred from naturally CO_2_-charged reservoirs^6,7^, through numerical modeling^8,9^ and investigations in lab experiments^5,10–14^. These laboratory experiments are often limited in predicting interactions and in-situ processes such as mixing with resident fluids, CO_2_ sorption and dissolution due to boundary conditions being simplified from real rock conditions^15^.

Typically, injection of CO_2_ is thought to lower the pH of the formation water inducing dissolution or precipitation reactions of minerals such as calcite^15–17^. Such physical and chemical interactions may alter the intrinsic flow properties of the rock^18,19^. Fracture permeability, for example, may effectively decrease through mineralization and swelling or increase through dissolution^19,20^.

Geophysical, e.g. seismic and pressure monitoring, and geochemical techniques, e.g. pH sensors, are available for the monitoring of storage sites^5,21–26^. Geochemical tracers can be suitable proxies for leakage detection by differentiating natural from injected CO_2_^17,27^. Such tracers include ion concentrations, stable isotopes such as $\delta ^{13}$C of the CO_2_ molecule, and noble gases^27–32^. These tracers can be used to identify the source of a fluid of interest through the computation of mixing and its temporal evolution^6,33–35^.

Specifically, noble gases allow inferring of critical transport mechanisms involved in the subsurface^34,36,37^. Due to their chemical inertness, they inform on the physical transport mechanisms and allow end-member calculations without the need to correct for chemical reactions. They are also frequently used as artificial tracers in injection experiments to constrain phase-partitioning, multi-phase flow and degassing under pressure changes (e.g.^38,39^).

In-situ injection experiments in natural settings have commonly been conducted on permeable deep storage reservoirs (e.g.^40^) or shallow aquifer systems^38,41,42^. In-situ studies in formations with permeabilities comparable to a caprock under reservoir conditions are sparse due to practical and experimental challenges^43^. A research program has been initiated in the underground Mont Terri rock laboratory (MTRL) to specifically address this knowledge gap^44^. The ambition is to provide quantification of the coupled physical and chemical processes controlling CO_2_ migration in a fault zone located in a clay formation.

The experimental setting aims to simulate a situation for which the CO_2_ stored in a reservoir reaches a fault crosscutting the caprock and accumulates overpressure: this is one of the most challenging situations for the safety and long-term containment of CO_2_. The MTRL offers a perfect natural, decameter scale geological environment where to test this situation. A thrust fault with variable thikness of c.a. 1 to 4 m is hosted in an almost impermeable claystone, known in literature as Opalinus Clay. The structure is easily reachable by boreholes drilled in a dedicated niche, where the MTRL management office offers and guarantees the possibility to perform long-term (multiple years) injections and subsequent monitoring.

In this paper, we present the results of a 14-month long injection of CO_2_-saturated water at the MTRL focusing on the geochemical analysis. The developed method allows in-situ observation of transport and geochemical mechanisms occurring at fracture scale. The results shed new light on the temporal scales involved in the migration of CO_2_-enriched fluid in the fault zone and quantify its interaction with resident formation water.

## Experimental context

### Injection site and experimental setup

**Figure 1:**
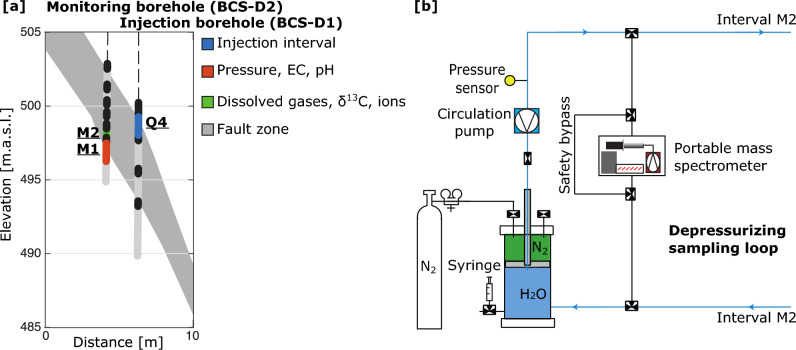
Experimental setup. (**a**) Injection and monitoring borehole setup in the Main Fault at the MTRL. The monitoring (M1 and M2) and the injection (Q4) intervals are marked. Interval M1 hosts the continuous monitoring system of physical and chemical parameters. Interval M2 is dedicated to sample fluids for stable isotopes, ion composition and on-site measurement of dissolved gases analysis through an internal depressurization system. (**b**) Sketch of the depressurization system connected to interval M2 where a portable mass spectrometer is set up. Figures modified after^44^.

The experiment (CS-D: Carbon Sequestration-Series D) was conducted in a new niche, that has been drilled from a recent extension of the MTRL, with the specific purpose to host the experiment^44^. The niche, c.a. 4 m wide-4 m high-18 m long, is positioned a few tens of m above the fault, almost perfectly perpendicular to its strike. The site was chosen given the presence of the well-known and well-studied fault zone in the caprock-like Opalinus Clay formation, with the installed boreholes intersecting that fault zone (Fig. 1a). During the characterization phase of the study site, the range of effective fault zone transmissivity was estimated between 1.8 $\cdot$ 10$^{-13}$ m$^{2}$ s$^{-1}$ and 9.2 $\cdot$ 10$^{-12}$ m$^2$ s$^{-1}$ resulting in permeability as low as 10$^{-21}$ m$^2$ and up to 10$^{-18}$ m$^2$ when specific features were pressurized at a pressure allowing substantial leakage^44^. Such features were identified in the collected core sample and analysis of borehole logs, and potentially connect hydraulically the injection and monitoring boreholes (Fig. 1a)^44–46^.

The second phase of the CS-D experiment featured a long-term injection of CO_2_-saturated fluid between June 2019 and August 2020. The injection was performed into the borehole interval Q4 (Fig. 1) at an imposed pressure of 4.5 MPa, just below the estimated fault leakage (or opening) pressure^44^. The injection was monitored through cutting-edge geophysical and geochemical methods, including a portable mass spectrometer^47^. The geochemical monitoring system comprised continuous monitoring of pH and electrical conductivity (EC), sampling for stable isotopes such as H_2_ and O_18_, the ion composition as well as the on-site measurement of dissolved gases including CO_2_ and noble gases (Fig. 1).

### Definition of the main fluid end-members

For the interpretation of the measurements in the monitoring borehole intervals M1 and M2 (Fig. 1), we identify three major fluid end-members with values derived in the “Methods” section “End-member definition”.

The first end-member is the resident ‘formation water’. Due to the installation of the borehole intervals, it was not possible to extract pure formation water, but an extensive database compiling geochemical data at the MRTL from previous experiments is available^48,49^.

The second fluid end-member is the ‘borehole water’, referring to the synthetic Pearson water used to fill the interval after completion. While the synthetic water has a similar ionic composition as the ‘formation water’, it significantly differs in terms of dissolved gases composition, being at equilibrium with atmospheric composition when filled. The first analysis performed with the mass spectrometer on-site on 23 April 2019 is considered as the baseline for the dissolved gas concentrations.

The third and last end-member is the ‘CO_2_-enriched injection water’. Constant CO_2_ concentration in the ‘injection water’ was maintained by continuously bubbling CO_2_ at a pressure between 2 and 3 MPa in a mixing tank, and periodically transferred to the pump for injection into the interval at 4.5 MPa. In addition to CO_2_, the injection fluid has been marked through pulse addition of Kr (see “Methods”). Furthermore, it is important to note that the volume of the monitoring interval is in the order of the volume of water injected (see “Results”). Therefore, fluid samples are a mixture of the fluid that is entering the monitoring interval with the fluids already present in the interval itself.

## Results

### Hydraulic response

**Figure 2:**
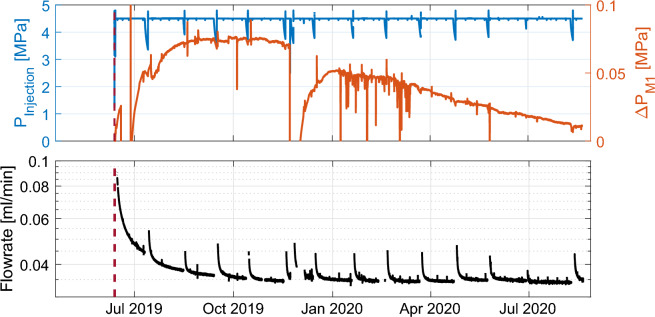
Hydraulic response. (**a**) Temporal evolution of injection pressure (blue line) and pressure change from background pressure (background approximately 0.1 MPa) in the monitoring borehole interval M1 (orange line) (see Fig. 1). (**b**) Flow rate of the water injection, which started on 12 June 2019 (dashed line).

Injection commenced on 12th June with the injection pressure being held constant at 4.5 MPa (blue line, Fig. 2a) until the 31st August 2020. About every 30 days, a hydromechanical test with shut-in/restart cycles was performed to infer potential permeability variation of the wellbore near-field (Fig. 2b).

A total of about 25 l of CO_2_enriched water was injected, which corresponds to an estimated 750 g of CO_2_ assuming a constant Henry coefficient of 3.4 · 10$^{-2}$ mol l$^{-1}$ atm$^{-1}$^50^. The injection flow rate decreased from an initial value of 0.1 ml min$^{-1}$ to approximately 0.035 ml min$^{-1}$ in October 2019 (Fig. 2b). Near steady-state flow was reached by the end of the experiment.

The injection activities did not end in August 2020, but continued in a different phase of the CS-D experiment until January 2021, with a stimulation phase and larger flow rates. Hence it is not possible to investigate the geochemical trends in a shut-in phase. The stimulation activity that occurred after August 2020 had an impact on both the observed geochemistry and the transmissivity of the fault zone. Given the complex behaviour during the stimulation activities, we leave the interpretation of post-stimulation activities for future work.

The pressure change in the monitoring interval M1, $\Delta$P, occurs almost instantly with the beginning of injection, revealing high connectivity of the fracture set. Pressure increased steadily (orange line Fig. 2a) until a maximum of 0.08 MPa pressure change that was reached in October 2019, corresponding to the time when the flow rate also stabilized (black line Fig. 2b).

Afterwards, the pressure monotonously approached the initial conditions. Disturbances in the pressure response are visible in the raw data as spikes or major temporary changes (Fig. 2a). Most of the signals were caused by operations in the tunnel: either failure of the sampling setup (e.g. July and November 2019) or poroelastic changes due to nearby activities (e.g. drilling of boreholes at a distance of 15–20 m along the fault zone, January to March 2020).

### Temporal evolution of CO_2_

The temporal evolution of dissolved gases was monitored with an on-site mass spectrometer that measures partial pressures^44,47^. The partial pressures are normalized to the initial baseline measurement in the monitoring interval and N_2_, i.e. $CO_2^*=\frac{CO_2/N_2}{CO_{2,i}/N_{2,i}}$ (equally for other gas species; for details see “Methods”).

**Figure 3:**
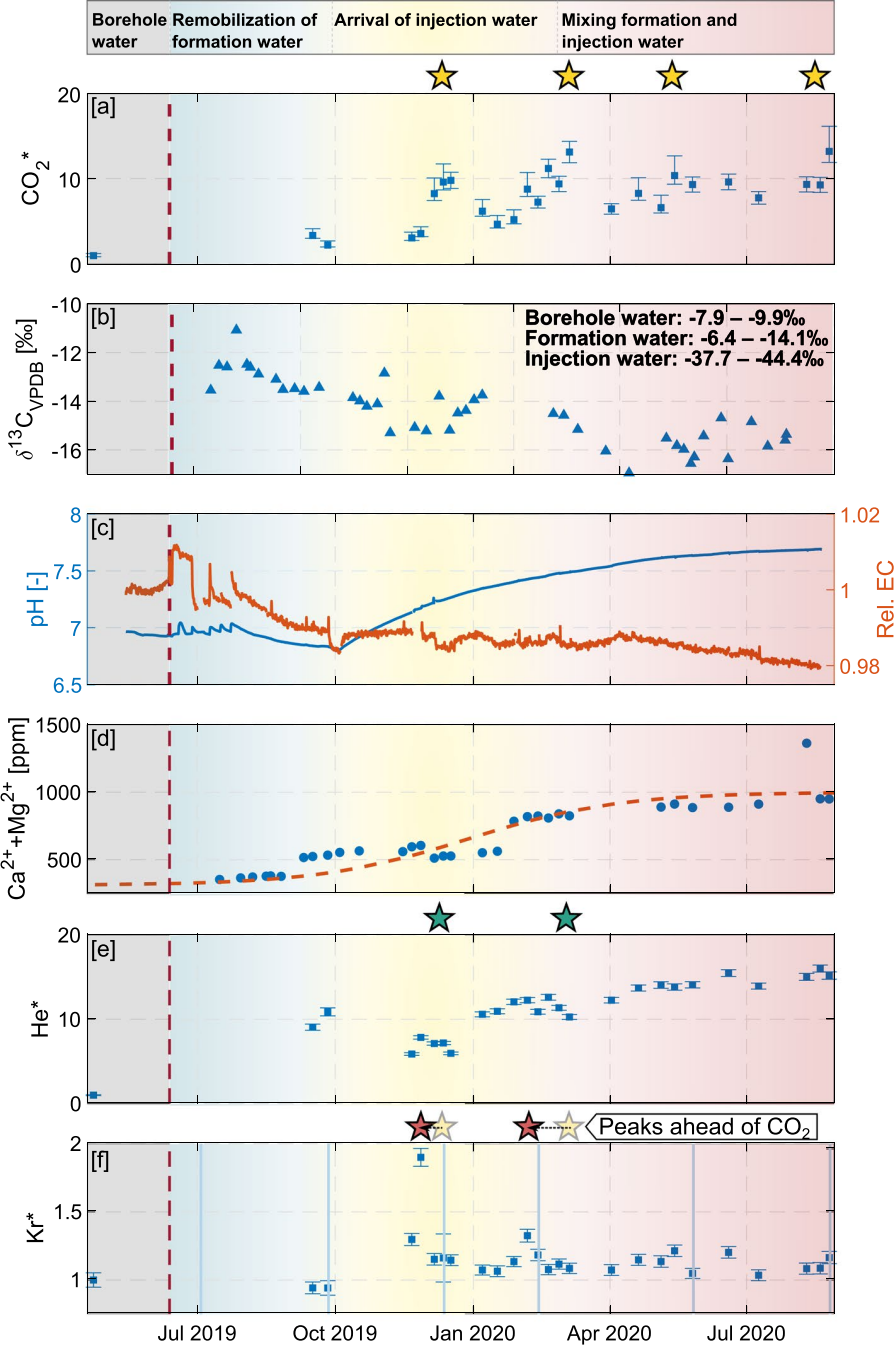
Evolution of physical and chemical parameters. (**a**) $CO_{2}^{*}$, CO_2_ relative to the first measurement ($CO_2^*=\frac{CO_2/N_2}{CO_{2,0}/N_{2,0}}$). (**b**) $\delta ^{13}$C$_{\textrm{VPDB}}$ with end-members noted. (**c**) pH (blue line) and EC relative to the mean of the month prior to injection (red line) (see Fig. 1). (**d**) [Ca$^{2+}$ + Mg$^{2+}$] with an idealized fitted curve in red. (**e**,**f**) Temporal evolution of dissolved noble gases He$^{*}$ and Kr$^{*}$, respectively. He$^{*}$ and Kr$^{*}$ defined as CO$_{2}^{*}$. The red line marks the start of injection on 12 June 2019. The blue lines in (**f**) mark the refill times of the injection tank. pH and EC were measured in interval M1, while the other data was measured in interval M2. The stars mark maxima or minima in the respective gases.

Dissolved CO_2_ content in interval M2 (Fig. 1) increased throughout the experiment reaching a value one order of magnitude higher than the initial baseline measurement (Fig. 3a). Two peaks in CO$_{2}^{*}$ (CO_2_ relative to the baseline and N_2_; see “Methods”) were observed with amplitudes of about 50% superimposed to the overall increasing trend (yellow stars Fig. 3a).

Previous studies have found that the formation water at the MTRL is enriched in CO_2_ and depleted in O_2_ through chemical and biological consumption^48^. Therefore, without any other extra information, considering the defined fluid end-members, the CO_2_ increase could result from either the formation water or the injection water.

However, the CO_2_ of the fluid end-members have distinct isotopic compositions, with formation and borehole water $\delta ^{13}$C ranging from − 14.1 to − 6.4‰ (all values V-PDB;), and injection water with significantly lower isotopic composition (− 37.3 to − 44.5‰). These differences in $\delta ^{13}$C values allowed the identification of whether injection water is causing the observed increase in CO_2_ (see “Methods” section “End-member definition”). The temporal evolution of $\delta ^{13}$C composition showed a global decrease (Fig. 3b). At the end of the injection period analyzed here, a $\delta ^{13}$C value of about − 17‰ was reached, which corresponds to the lowest value ever measured at the rock laboratory (Fig. 3b). $\delta ^{18}$O of the CO_2_ was also sampled, but did not reveal significant trends (Supplementary material Fig. S1).

A simple mixing calculation reveals that the relative content of the ‘injection water’ in the monitoring borehole progressively increases throughout the experiment to reach a maximum of approximately 20% at the end. The only end-member in the system with low $\delta ^{13}$C is the injected fluid (range − 37.7 to − 44.4‰). Hence the joint analysis of CO_2_ and $\delta ^{13}$C unambiguously reveals the contribution of ‘CO_2_-enriched injection water’ at the monitoring borehole.

### Evolution of physical and chemical parameters

Continuously measuring sensors located in monitoring interval M1 revealed a decrease in pH from about 7 at the beginning of the experiment to a minimum of 6.8 observed on 4 October 2019. Afterwards, the pH began increasing and reached a slightly basic value of 7.7 at the end of the experiment (Fig. 3c).

Electrical conductivity (EC) decreased after the start of injection until October with a relative change of 2% (Fig. 3c). After October 2019, EC decreased with about 1% change for the remaining 12 months until the end of the experiment. We note the synchronicity between the sudden changes in EC evolution with the one in pH in October 2019. Interestingly, this also corresponds to the time at which the injection flow rate reaches near steady-state conditions (Fig. 2b).

The evolution of the major ion concentrations is also constrained by mixing between the main geochemical end-members, i.e. borehole water, formation water and injection water, but can additionally be subject to chemical reactions. When end-members for the measured ions were different, mixing trends were observed throughout the experiment, from a signature of the ‘borehole water’ towards a mixture of the ‘formation water’ and the ‘injection water’ (Supplementary material, Fig. S2). A simple mixing calculation based on K$^+$ trend shows that injection water and formation water together account for 70% of the water in monitoring interval M2.

The sum of calcium and magnesium also follows this ideal mixing pattern, represented by a sigmoid function fitting (dashed line in Fig. 3d). It becomes apparent that additional systematic changes are superimposed to the mixing. A first increase occurs in September 2019 possibly indicating the arrival of the injected fluid (Fig. 3d). This increase appears a few weeks before the increase in pH. On the one hand, this early arrival could be linked to the water injected during the characterization tests^44^. On the other hand, since no other parameter exhibits such a high content of the injection water at this point in time, the change could be caused by chemical reactions. There is a decrease of [Ca$^{2+}$ + Mg$^{2+}$] around January 2020 which again could be linked to chemical reaction. Given the monitoring setup, it is difficult to identify the potential reactions causing these changes.

Gaps in measurements of CO$_{2}^{*}$, He and Kr are due to technical issues with the mass spectrometer, due to water entering the instrument. The water inflow was caused to fail by the fact that the semi-permeable membrane did not withstand the high interval pressure. More technical details of the solution to this aspect is discussed in^51^.

In Fig. 3d one data point, after July 2020, exceed 1000 ppm in Ca + Mg concentration. It can be argued that it is the effect of dissolution of calcite, despite no significant fluctuations in pH are observed in that time period. However this observation concerns only a single data point in the whole time series, and we interpret this as an outlier due to sampling and/or measurement uncertainties.

### Variations in noble gases

He$^{*}$ (defined equally as CO$_{2}^{*}$; see “Methods”) showed an increase by more than one order of magnitude over the course of the experiment (Fig. 3e). This increase is caused by the ‘formation water’ end-member which is the only one with high He content. It appears to be remobilized in response to the injection pressure. This remobilization of formation water resulted in an increase in partial pressure of one order of magnitude until October 2019 since the start of the long-term injection. This increase is followed by a temporary decrease in He$^{*}$ which advances and overlaps with periods of time of increased CO$_{2}^{*}$ and Kr$^{*}$ (green stars Fig. 3e). In the last months, a pseudo-constant level is reached approximately 15 times greater than the atmospheric baseline suggesting steady mixing of the different end-members.

Kr$^{*}$ displays smaller overall changes compared to the other gases. Nevertheless, two peaks (red stars Fig. 3f) can be identified compared to the initial partial pressure. The most significant one occurred at the end of November 2019, and a minor one in February 2020. In contrast to CO_2_, for which saturation in the injection water was kept constant, Kr was only added during tank refilling (see “Methods”). Apart from the peaks arising from the ‘injection water’, the differences between the end-members are not significant enough to result in long-term changes.

The peaks of Kr$^{*}$ occur 2 and 4 weeks prior to the corresponding peaks in CO$_{2}^{*}$. This temporal shift can be interpreted as differences between non-reactive and reactive transport. For all gases, the decreasing flow rate during the progress of the experiment increases the impact of dispersion on the peaks leading to a broader peak shape.

**Figure 4:**
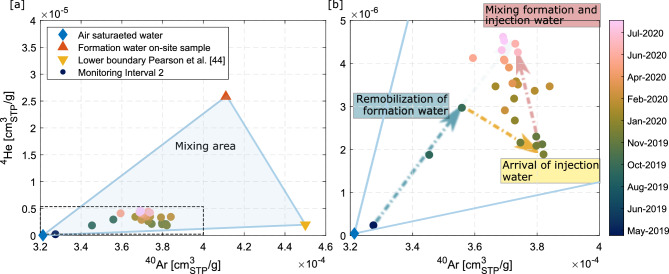
Noble gas mixing. (**a**) The He and Ar concentrations fall within the mixing area constrained by the atmospheric composition of the borehole water, a sample taken from formation water on site and the lower boundary of previously observed Opalinus clay noble gas data^48^. Dots are colour-coded by sampling date. (**b**) Zoom into the temporal evolution of He and Ar concentrations [(corresponding to the dashed area in (**a**)]. In the first step, concentrations reveal the mixing and/or remobilization of formation water for the first four months (blue arrow). Then, the arrival of the injected front fosters a sharp change towards lower values of He and higher values in Ar (yellow arrow). Finally, He and Ar concentrations progressively come back to the initial mixing line ratio (red arrow).

To compare the noble gas trends to measurements of the formation water, we derive the concentrations through Henry’s law (see “Methods”). He concentrations showed variations by more than a factor of ten, whereas Ar concentrations only of about 15% during the CS-D long-term injection phase. The concentrations of He and Ar lie within a mixing area of the main end-members (Fig. 4a): (1) the borehole water, (2) the injection water, both with atmospheric values, and the (3) formation water, being defined by a wider range of non-atmospheric concentrations observed in an on-site sample in an interval with no pressure perturbation and during previous experiments^48^ (see “Methods” ).

The He and Ar concentrations during the first four months of the experiment define a mixing line between atmospheric concentrations and a typical non-atmospheric composition in the expected range (Fig. 4b). At the end of the experimental phase, a mixing calculation based on He concentrations results in a share of the formation water at the monitoring interval of 45 ± 5%. The error mainly arises from systematic uncertainty of noble gas concentrations of the pre-existing formation water being used to determine the mixing ratios (blue arrow Fig. 4b). The progressive increase in admixing formation water is interrupted by the arrival of the injection water in December 2019, which is identified by its lower He concentration (yellow arrow Fig. 4b). After that time, He and Ar concentrations stabilize towards the initial mixing line, whereby an equilibrium between the different fluids contributing is approached (pink arrow Fig. 4b).

## Discussion

One of the main risks involved in deep geological storage of CO_2_ relates to leakage through the caprock^5^. Potential caprock formations, such as clay-rich formations, typically have low permeability, strongly limiting fluid migration. However, preexisting fractures and faults, or even hydraulically-induced fractures created during high-pressure CO_2_ injection, may allow CO_2_ to migrate toward shallow aquifers. CO_2_ migration is mainly driven by buoyancy effects and by the pressure gradient between the storage reservoir and the overlying formations. Its temporal and spatial scales are dependent on the transport properties of the main permeable structures and the chemical reactions between the CO_2_, the resident fluid, and the rock formation. The CS-D experiment provided the first in-situ insight into processes controlling fluid migration across a caprock analogue and their short-term temporal evolution at the scale of a few meters.

### Fluid migration through the fault zone and mixing

**Figure 5:**
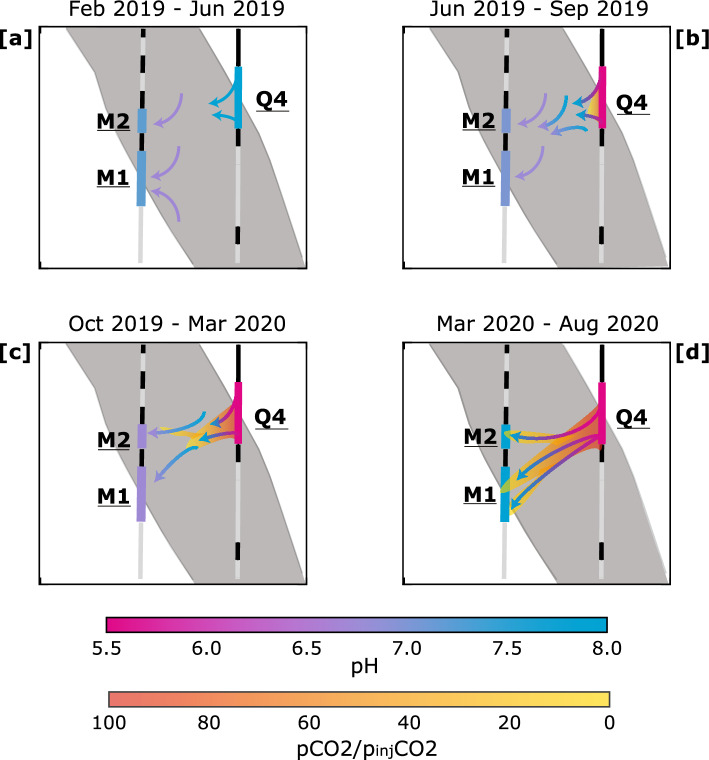
Conceptual model of mobility of CO_2_ captured in four different times: (**a**) During the preliminary injection tests we observe a clear pressure signal, between the injection interval Q4 and the monitoring intervals M1 and M2) CO$_{2}^{*}$, interpreted as in-situ water pushed towards the monitoring interval. (**b**) In the first few months of injection activities we observe a decrease of the pH. This is interpreted as more in-situ water being pushed from the rock toward the monitoring interval, allowing for further dilution of the synthetic water and a further decrease of the pH, our interpretation for this phase is that the CO_2_ has not traveled far from the injection point, and it has then not reached the monitoring borehole. (**c**) Breakthrough time: in October 2019, the trend change of pH is interpreted as injection water reaching the monitoring point, with an extremely low amount of CO_2_ with a pCO_2_ much below the one at injection arrive in M2. (**d**) Both the CO_2_ content and the pH increase, confirming that the breakthrough has actually occurred but not all the fluid has been replaced.

Our interpretation of the mobility of injected water, based on combined observation on pressure evolution, pH and CO_2_ detected by the mass spectrometer, is graphically illustrated in Fig. 5.

We found that the fault zone does not act as a straight pathway for the migration. In contrast, the fracture network within the fault zone seem to constrain migration through preferential paths that may not follow the main direction of the fault zone. Such permeable fractures were suggested to be present during the characterization phase of the experimental site. At the scale of the experiment, i.e. a distance of a few meters between injection and monitoring intervals, the fractures within the main fault zone allow for the transfer of the injected CO_2_-enriched water.

Based on the peaks in CO_2_ (yellow star, Fig. 3a), CO_2_ migrated a distance of 2.23 m on the timescale of 150–180 d. This corresponds to a fluid velocity of 1.5$\cdot$10$^{-7}$ m s$^{-1}$. The arrival of the CO_2_ plume at the monitoring borehole is further identified by the combined analysis of EC, pH, ion concentrations, and, especially, $\delta ^{13}$C and Kr (Fig. 3b–f).

The estimated fluid velocity seems too high for the permeability/transmissivity and porosity values found from core sample and during the in-situ characterization phase: between 10$^{-19}$ to 10$^{-21}$ m$^2$ and 4% to 11%, respectively^44,46^. The highest observed transmissivity value of 9.2 $\cdot$ 10$^{-12}$ m${^2}$ s$^{-1}$ (roughly corresponding to a permeability of 6.5$\cdot$10$^{-19}$ m$^2$) was found by Zappone et al.^44^ upon reaching the fault leakage/opening pressure. Even using this value, a transport calculation would yield a longer time for the fluid to travel the 2.23 m distance between the injection point and the monitoring point than observed. This would be a very strong assumption, as there is a gradient of 3.5 MPa between the injection point and the monitoring point, hence the entire distance would not be at a pressure above the opening of small fractures.

The estimate of permeability considered a homogeneous porous medium with a thickness as large as the injection interval (1.4 m). However, if we assume that the flow is occurring only on very small, limited fractures, the observed fluid velocity would be more consistent with the estimated non-stimulated in-situ transmissivity (10$^{-13}$ m$^2$ s$^{-1}$). For this transmissivity, and with the estimated fluid velocity of 1.5$\cdot$10$^{-7}$ m s$^{-1}$, the effective flow should only occur in a limited thickness of about 1 to 3 mm, for a porosity of 11% and 4%, respectively ($h=\frac{T}{\rho _w g}\frac{\Delta P}{L}\frac{1}{\phi v}$, where *T* is the estimated transmissivity, $\rho _w$ is the water density, *g* is the gravity, $\Delta P$ is the pressure difference, *L* is the distance between the injection and monitoring point, $\phi$ is the porosity, and *v* is the estimated fluid velocity). This calculation reveals the key role of preexisting fractures in enhancing fluid migration velocities through the caprock.

Considering the contrast in permeability between the fracture and the matrix, the contribution of matrix diffusion in controlling the time of the arrival of the front is negligible at the temporal scale of the given phase of the CS-D experiment.

For the first two peaks in CO_2_, we can associate peaks in Kr which systematically occur before the arrival of CO_2_ (2 and 4 weeks, respectively; red stars in Fig. 3f). The peaks in CO_2_ and Kr are linked to the refilling operations of the mixing tank that temporarily increased the concentrations of both gases at the injection point (blue lines, Fig. 3f). Besides the difficulty in identifying the time of the actual peak concentration due to the relatively low sampling frequency for gas measurements, the difference in breakthrough times appears to be consistent between the different refilling operations (blue lines Fig. 3f).

Two explanations for the differences in arrival times of CO_2_ and Kr are plausible. On the one hand, the diffusion properties of the two species are different. Although CO_2_ has a larger diffusion coefficient, the peaks occur later than the Kr peaks (D$_{Kr}$ = 1.12·10$^{-9}$ m$^2$ s$^{-1}$ vs. D$_{CO_2}$ = 1.46$\cdot$10$^{-9}$ m$^2$ s$^{-1}$^52^). Previous experiments suggested that the effective diffusion is dependent on the molecular volume of the species and that a higher diffusion coefficient can be disadvantageous for their mobility^53,54^. In detail, larger and/or less diffusive atoms and molecules tend to remain on the transmissive preferential pathways of advective transport and do not enter stagnant dead-end pores as easily as smaller ones^53,54^. These effects could be at play at the site of the CS-D experiment and explain the earlier arrival of Kr.

On the other hand, the difference in arrival time could be caused by chemical retardation of the CO_2_. Retardation might result from fluid-rock or fluid–fluid interactions and mixing of fluids with contrasted chemical composition inducing processes such as adsorption, swelling, dissolution, precipitation, speciation, and ion exchange reactions^55–59^. Chemical reaction of CO_2_ could be one of the reasons for the differences in arrival time even if we observe a constant pH level above 7. Indeed considering the pH values monitored, dissolution is unlikely to be dominant. However, the pH data are provided at a monitoring intervals at a distance of about 2.5 m from the injection interval, while the reactions are expected near the injection well itself (and consequently the drop in pH). In fact, the pH of the injected water has been observed to be  5.5 (as highlighted in the conceptual model in Fig. 5). The temporal evolution of He concentrations at the monitoring interval (Fig. 4) revealed mixing between different geochemical end-members which may favour such chemical reactions. We have quantified mixing between the injected fluid and the formation one based on the analysis of complementary tracers. We specifically used the $\delta ^{13}$C fraction and conservative noble gases such as He and Ar (naturally enriched in the formation water) and Kr that we added in injection fluid. All those tracers provided complementary information into the origin of the water collected at the monitoring interval. He is a sensitive tracer that has been previously used to quantify mixing with formation water and fluid remobilization during subsurface operations^34,35,60,61^. In the CS-D long-term injection phase, injected fluid is under-saturated in He with respect to the formation water and He still acts as a valuable natural tracer. The changes in ion concentrations are the most direct observation of potential induced chemical reactions (Fig. 3d). Also the [Ca^2+^+ /Mg^2+^] ratio showed variability during the experiment which could be caused by dissolution of calcite, which constitutes up to 90% of the carbonate content^48^. Other reactions may have occurred at different times, but there is not enough indication to draw meaningful conclusions.

The migration at the scale of the CS-D experiment may seem relatively fast since the flow paths are minor fractures. However, the likelihood that such fractures are extending over a long distance is very small. Extending this concept to a large-scale CO_2_ sequestration site implies that the likelihood that the CO_2_ can travel through a caprock, or even along a fault within the caprock itself, is very low, despite the possibility of locally fast transfer over limited distances.

### Monitoring of CO_2_ leakage through caprocks: lessons learned, opportunities and challenges

Early and reliable monitoring of leakage through a caprock is a major challenge for carbon sequestration operations. Prevention relies on the monitoring of the CO_2_ storage site through joint geophysical and geochemical instrumental setups. In the long-term injection phase of the CS-D experiment, geochemical monitoring was essential to resolve the main coupled physical and chemical processes. Further, the range of parameters measured proved valuable and sole reliance on CO_2_ measurements during monitoring can be misleading since CO_2_ is ubiquitous in the environment and can exhibit large natural variability^62^. Therefore, the applied multi-tracer approach was necessary to allow differentiation between the different CO_2_ sources and further allowed resolving temporal scales of the ongoing processes at the scale of the experimental setup.

The results presented for the CS-D experiment are relevant to the proper understanding of CO_2_ leakage pathways through a fault zone in a caprock. While the spatial and temporal scales are far from the full-scale of a CO_2_-sequestration site, the results can help to further improve the monitoring of caprock failure at large scales.

The high He concentrations of the formation water are a typical geochemical feature of terrestrial fluids in the deep subsurface. It is therefore considered an adequate candidate to be used as a natural tracer in a multitude of applications involving subsurface operations. This is also likely to be effective for supercritical/gaseous CO_2_, which will based on phase partitioning calculations, will strip noble gases including He from the formation water^32,63^. The use of He is supported by a study at the Weyburn EOR/CCS site^36^, where He isotopes were used to prove that injected CO_2_ was not contaminating shallow ground and surface water. Further, fast effective diffusion of larger noble gases allows them to be used as early warning tracers to prevent fluid leakage^54,64^. The current results confirm that a combination of chemically different tracers and higher sampling frequency leads to an improved description of the processes.

We observed changes in the ion composition that could serve as a proxy for identifying leakage through the caprock as suggested in other work^17^. The reactions observed through calcium and magnesium could be used for leakage monitoring. At other sites, the functionality of ions in monitoring may differ depending on the composition of the host rocks. pH did not show a significant decrease in the injection experiment. On long-term exposure to highly concentrated CO_2_, acidification may occur more pronounced as the buffering capacity of the host rock could become exhausted. The development of pH may then have a more pronounced monitoring significance for a CO_2_ storage site.

We also used $\delta ^{13}$C to quantify mixing. We took advantage of contrasting values between the injection and formation water completed in other studies^25,29,65^. However, it is known that carbonate dissolution associated to the injection of CO_2_ may impact observed $\delta ^{13}$C (e.g.^66–68^). This could result in dissolved inorganic carbon with a $\delta ^{13}$C value that covers the signal of the injected CO_2_. In the case of the Opalinus Clay, calcite $\delta ^{13}$C is approximately 0‰^48^. The observed variability in $\delta ^{13}$C suggests that the chemical reactions are affecting the composition of $\delta ^{13}$C (Fig. 3), which in turn may lead to underestimation of the CO_2_ content. Hence, reactions tend to reduce the reliability of stable isotopes as a tracer. This is in line with conclusions from^29^, who argued that differences in $\delta ^{13}$C of at least ± 10‰ between end-members are required to use $\delta ^{13}$C in geochemical monitoring schemes.

One drawback, however, is that the approach employed at CS-D required a large number of gas and fluid samples. In the CS-D experimental setup, this sampling was possible thanks to the unique configuration (packed intervals) and very limited spatial extent for the fluid to travel. The same may not be true for a full storage site, and it may only be possible when a leakage pathway is already identified by other methods, such as active or passive seismic characterization, as well as geomagnetic methods.

## Conclusion

The pathways of fluid migration in a fault zone in the specific case of low permeable formation are far from being trivial. We conclude that the fractures present in a fault zone can indeed foster fluid migration, but in the configuration of the CS-D experiment, fluids seem to travel across rather than along the fault zone. The results highlight fundamental mechanisms by which the fluid transfer may occur: even in very low permeable formations (i.e. caprocks), the presence of fractures strongly affects the fluid migration, much more than the diffusive front. This conclusion may only be true for such a small scale as that of the CS-D experiment, since the fractures can be expected to be of limited scale. Therefore, up-scaling of the fluid velocities to large scales could be misleading. In reality, the presence of many fractures can actually further hinder the flow through a fault zone, as it would lead to a more tortuous pathway for the CO_2_ to escape. The fact that single fractures are dominating the fluid migration is actually a further proof that CO_2_ storage is a viable solution to reduce emission to the atmosphere.

The injection was carried out at relatively high pressure, but still below the pressure that would cause further permeability increase. Hence, the long-term injection phase for the CS-D experiment highlights how the fluid can leak through a fault zone that is not exposed to reactivation (and hence with very limited induced permeability/porosity changes). If leakage were to occur through small fractures the amount would be limited both in volume and mass.

The success of the geochemical monitoring presented here makes a strong argument in the favor of joint multi-tracer geochemical and geophysical methods. Such a combination would allow critical assessment of how CO_2_ injections might affect the natural fluid dynamics. The multi-tracer approach would allow resolving coupled hydraulic and transport processes relevant at the investigated spatio-temporal scales. As in the case we presented here, noble gases can play an important role in providing an early warning of CO_2_ leakage. C isotopic composition is a key factor in identifying mixing sources. The success is also strongly dependent on the prior knowledge of the end-members and on the addition of tailored tracers to make the injected CO_2_ identifiable against the natural background.

From a technical perspective, the developed monitoring scheme and setup with a well sampling line is a successful proof of concept for the application of on-site dissolved gas analysis under high pressure and low permeable environments. The setup can be adapted to specific conditions to track chemical signals at temporal and spatial scales relevant to early warning systems.

## Methods

### Study site

This study was carried out during the second phase of the “CS-D” experiment (Carbon Sequestration-Series D)^44,45^. The study targets the Opalinus Clay Formation of the Mont Terri rock laboratory (MTRL) located in the sedimentary rocks of the Swiss Jura^69^. The MTRL is run by an international consortium, managed by the Swiss Federal Office of Topography (swisstopo), and mainly aims to investigating nuclear waste disposal in real conditions^69^.

The research galleries are around 280 m below the surface. With a permeability as low as 10$^{-21}$ m$^{2}$ and self-sealing properties the Opalinus Clay has been a target to host nuclear waste disposal^56,69^ but is also an easily accessible analogue for a caprock in CO_2_ storage projects^14^.

A new research gallery (‘Niche CO_2_’), was excavated and installed for the experiment. Near the niche, the Opalinus Clay Formation is intersected by the SSE-dipping fault, the Mont Terri Main Fault (MTMF) (Fig. 1). The fault served as the experimental target for the injection of the CO_2_-enriched water as an analogue for a typical fault zone.

Zappone et al.^44^, Wenning et al.^46^ have described the geometry and hydraulic properties of the fault zone. At the experimental site, the fault has varying thickness of 1.5 to 3 m with a mean orientation striking N053$^\circ$ and a dip of 46$^\circ$ SE^44^. Based on core analysis and inter-borehole geophysics, the experimental volume was described and imaged including a complex preexisting fracture network. The internal damage zone is very heterogenous, typically not cross-correlated between boreholes, with μm-thin fractures, making up distinct regions of shaly clay, two types of fault gouge and calcite veins^46^. Based on borehole hydraulic testing experiments^44^, have revealed efficient connectivity for pressure diffusion throughout the monitoring network.

### Experimental setup

The spatial and temporal scales of this experiment were designed to allow observation of the key processes in a relatively high pressure and low-permeability environment.

A system of seven boreholes for injection and monitoring purposes was drilled to intersect the MTMF, during the first phase of the CS-D experiment^44^. The borehole network consists of one injection borehole and six observation boreholes. The injection borehole (BCS-D1) is equipped with a 4-fold packer system which isolates different intervals within the MTMF (Fig. 1). The fluid monitoring borehole (BCS-D2) is parallel to the injection borehole with a distance of approximately 2 m and is equipped with six packers (Fig. 1). The boreholes were filled with synthetic water after installation.

The intervals M1 and M2 of the fluid monitoring borehole BCS-D2 (Fig. 1a) are equipped with circulation systems allowing for geochemical monitoring and frequent fluid sampling. Boreholes BCS-D3 to BCS-D6 are dedicated to geophysical monitoring as described in^44,45^.

#### Injection setup

The injection system is made up of an ISCO syringe injection pump with a 0.5 l reservoir for moderate pressure injection over long periods. The ISCO reservoir was automatically refilling from a connected mixing tank with a volume of 10 l. The mixing tank was filled with injection water, CO_2_ and the desired trace gases. The mixing tank was connected to a circulation pump and pressurized with a CO_2_ bottle to achieve the desired saturation pressure.

The pressure in the mixing tank was monitored and maintained between 2 and 3 MPa. The ion content of the injection water was adjusted to the formation water as inferred from^69^. The noble gas Krypton (Kr) was added as a conservative gas tracer due to its inertness and its low background concentrations in the formation water^48^. Both gases were added during six refill operations of the mixing tank.

#### Geochemical monitoring: Inline sampling

Interval M1 of the monitoring borehole was equipped with a circulation pump to allow for frequent fluid sampling and in-line monitoring from the experimental niche. Samples can be collected at system pressure by closing off in-line sample containers. M1 is further equipped with the in-line pH meter ‘Hamilton Polilyte Plus, pH range 0–14’ and the electrical conductivity (EC) meter ‘Hamilton Conducell, 1–300,001 μS cm$^{-1}$’.

#### Geochemical monitoring: depressurized sampling

Interval M2 of the monitoring borehole was connected to a circulation pump and a piston tank and by-pass lines (Fig. 1b). Under regular conditions, the circulation line was held at system pressure (i.e. the same pressure as the interval M2 at depth). A reduced pressure was needed for gas sampling. The bypass line allowed isolation of the piston tank from the main interval. Then, depressurization of the piston tank allowed for sampling at a reduced pressure of 1.5 bar. After gas and fluid sampling, the piston tank was re-pressurized to a value similar to the one on the interval via a N_2_ bottle acting on the piston (hence not mixing with the internal water). Only after the re-pressurization, the circulation could be restored with the interval M2 at depth. Sampling was conducted on a regular weekly to bi-weekly basis.

The fluid tank of that circulation line has a volume of 12 l. Therefore, the fluid in the tank and the respective samples represent a mixture of the fluids in interval M2 over time.

Additionally, a single sampling was performed in a non-pressurized interval (uppermost one in the borehole BCS-D2) to create a baseline for the noble gas mixing analysis. The sample was taken in a copper tube closed off by cold welding after interval circulation with an external pump for few hours (to avoid sampling the fluid lines instead of the interval itself.

##### On-site mass spectrometry

The depressurizing set-up described above made it experimentally possible to analyse dissolved gases sampled via a membrane inlet system, which in common design does not withstand pressures in the MPa range. Once the system was depressurized, the fluid was circulated in a closed loop through two parallel extraction membrane modules (3M$^{\textrm{TM}}$ Liqui-Cel$^{\textrm{TM}}$ MM-0.5-1x1 Series), such that the dissolved gases reach equilibrium with a gas head space. The gas analysis was performed with a portable Gas-Equilibrium Membrane Inlet Mass Spectrometer (GE-MIMS) (Fig. 1^47^).

The equilibrated gas in the headspace is connected to the quadrupole mass spectrometer where its composition is analyzed on the intensities of the m/z-ratios for He, N_2_, O_2_, Ar, CO_2_ and Kr. An analytical cycle lasts six minutes including purging the line connecting the sample port with the mass spectrometer before measurement. The intensity of a gas is calibrated against a standard gas by peak-height comparison to calculate partial pressures in a gas mixture. As standard gas tunnel air was sampled providing atmospheric baseline. When CO_2_ and Kr intensities were significantly above previous measurements due to the injection activities in the tunnel, the standard sample of the previous sampling day was used.

The gas consumption of the mass spectrometer is approximately 0.1 ml min$^{-1}$^47^. To maintain equilibrium in the gas headspace, ideally, a flow rate of more than 1 l min$^{-1}$ through the membrane module is desired. Such an amount of water, and respectively the dissolved gas, was not available given the small total gas amount available for measurement. To guarantee stable detection of dissolved gases, analysis was not conducted for longer than one hour per sampling day, which is equivalent to three to six analytical cycles. This keeps the gas consumption low to maintain equilibrium within the gas headspace of the membrane modules. A daily measurement is then calculated as the mean of the conducted analytical cycles.

All values were normalized to the measured partial pressure of N_2_ to limit uncertainty in partial pressure due to variations in pressure within the sampling system. Since N_2_ was not part of the tracer tests and is typically of atmospheric origin, its measured variation is typically only dependent on inlet pressure at the extraction membrane and machine-related variation. We use this effect to define the normalized value of a dissolved gas species, X, as $X^*=\frac{X/N_2}{X_{i}/N_{2,i}}$, hence CO_2_ normalized by N_2_ and relative to the initial, atmospheric measurement X$_{i}$.

To establish the baseline conditions in the monitoring interval, the interval was sampled on 24 April 2019 prior to injection. Even though the depressurization system was not yet installed, the observed N_2_ intensities verified that measurements were performed within the correct pressure range.

From the measured partial pressures we estimated the concentrations of He and Ar through Henry’s law^50^. An average temperature of the laboratory tunnel of 18.5 $^\circ$C and an atmospheric pressure of 1013.25 mbar were used for deriving the solubilities.

##### Laboratory based fluid analysis

Fluid samples for subsequent laboratory analysis were collected from the depressurized line (Fig. 1b). To determine the carbon isotope composition of the dissolved inorganic carbon, 1 to 2 ml of water were injected immediately after sampling in 12 ml septum capped borosilicate vials containing 150  μl of 85% phosphoric acid and previously flushed with high purity He to convert the dissolved inorganic carbon completely to CO_2_. The generated CO_2_ was analyzed on a Gasbench II coupled to a Delta V mass spectrometer (Thermo Fisher Scientific, Bremen). Solutions of Na-bicarbonate of known carbon isotope composition were used for standardization.

Three samples for stable isotope analysis were taken and analyzed per sampling date and their mean and standard deviation is used as value and error, respectively.

The ionic concentrations were measured with a Dionex DX-120 Ion Chromatograph (Thermo Fischer). For the anions, the concentrations in fluoride, chloride, nitrite, bromide, nitrate, phosphate and sulphate were quantified. Concentrations in lithium, sodium, ammonium, potassium, magnesium, calcium as well as strontium was measured for the cations.

### End-member definition

#### $\delta ^{13}$C

The formation water has been observed to be of variable $\delta ^{13}$C composition, ranging from − 14.1 to − 6.4‰ (all values V-PDB;) (this study and^70,71^). These previous studies suggested that the isotopic signature of the CO_2_ in the formation water results from ‘equilibrium between aqueous solution species and diagenetic carbonate minerals’^70^.

The borehole water end-member falls into that range of values with an isotopic composition measured in two sampled of − 9.9‰ and − 7.9‰, respectively,

The injection water derives its $\delta ^{13}$C signature mainly from the CO_2_ that is bubbled through the injection water. That labels the injection water with CO_2_ having a significantly lower isotopic composition (− 37.3 to − 44.5‰).

Considering the similarity of the borehole water and the formation water, they can be considered as one mixing component. In the mixing calculation, the average of the measurements was used to define the value of that combined end-member to − 9.0 ± 0.5‰.

#### Noble gases

Helium (He) is ubiquitous in the subsurface, originating from atmospheric exchanges or radiogenic production^72^. Also at the MTRL, previous studies showed high He concentrations in the formation water due to radiogenic production from the Opalinus Clay^48^. He concentrations from different locations in the MTRL ranged from 2 $\cdot$ 10$^{-6}$ cm$^{3}_{STP}$ g$^{-1}$ up to 1.3 $\cdot$ 10$^{-4}$ cm$^{3}_{STP}$ g$^{-1}$, which are significantly higher than water in air-saturated water (ASW, 4.6 $\cdot$ 10$^{-8}$ cm$^{3}_{STP}$ g$^{-1}$ at $10\,^\circ \text{C}$)^48^. High values of total dissolved He are the result of radiogenic accumulation of $^{4}$He. The wide range of observed He values at the study site reflect variability in groundwater residence time and recharge conditions. Ar concentrations (3 $\cdot$ 10$^{-4}$ cm$^{3}_{STP}$ g$^{-1}$ to 4.5 $\cdot$ 10$^{-4}$ cm$^{3}_{STP}$ g$^{-1}$) were, if at all, only slightly enriched against ASW (3.8 $\cdot$ 10$^{-4}$ cm$^{3}_{STP}$ g$^{-1}$)^48^. The lower values define the lower range of the formation water shown in Fig. 4.

The upper limit of the range in He and Ar concentrations is further constrained by a sample taken during this experiment analysed by conventional mass spectrometic methods (see^73^). This sample resulted in He concentrations of 2.6 $\cdot$ 10$^{-5}$ cm$^{3}_{STP}$ g$^{-1}$ and Ar concentrations of 4.1 $\cdot$ 10$^{-4}$ cm$^{3}_{STP}$ g$^{-1}$. The $^{3}$He/$^{4}$He was measured to 1.47 $\cdot$ 10$^{-7}$ which is significantly lower the the atmospheric ratio of 1.34 $\cdot$ 10$^{-6}$.

Kr shows little variability in environmental fluids, is almost completely of atmospheric origin and has naturally low concentrations in groundwaters. Also in the rock laboratory, Kr was previously observed to be at or slightly below ASW concentrations^48^.

#### Ion concentrations

For the mixing calculation based on potassium, the end-members were solely defined through sampling. For the formation water composition sampled from monitoring interval M1 were assumed to be representative since it had been equilibrating with the formation over several months before the start of the injection experiment. This results in values of 0.056 ± 0.008 meq l$^{-1}$ for the borehole water, 0.009 ± 0.003 meq l$^{-1}$ for the formation water and 0.002 ± 0.001 meq l$^{-1}$ for the injection water.

### Supplementary Information

Supplementary Figures.

## Data Availability

All data related to the current study are available in the ETHZ Research Collection database (https://www.research-collection.ethz.ch/handle/20.500.11850/602648).
