# Supplementary material for: In-situ experiment reveals CO2 enriched fluid migration in faulted caprock
Source: Sci Rep. 2023 Oct 9;13:17006. doi: 10.1038/s41598-023-43231-6 (PMC10562487; doi:10.1038/s41598-023-43231-6)
Supplement: Supplementary file 1 — Supplementary Figures. [file 41598_2023_43231_MOESM1_ESM.pdf]

Supplement to: Geochemical Insight into CO_2 Migration in a Faulted Caprock

δ^{18}O

δ^{18}O was measured analogously to δ^{13}C (Fig. 1). Differences in the end-members were not significant enough to allow for mixing calculations.

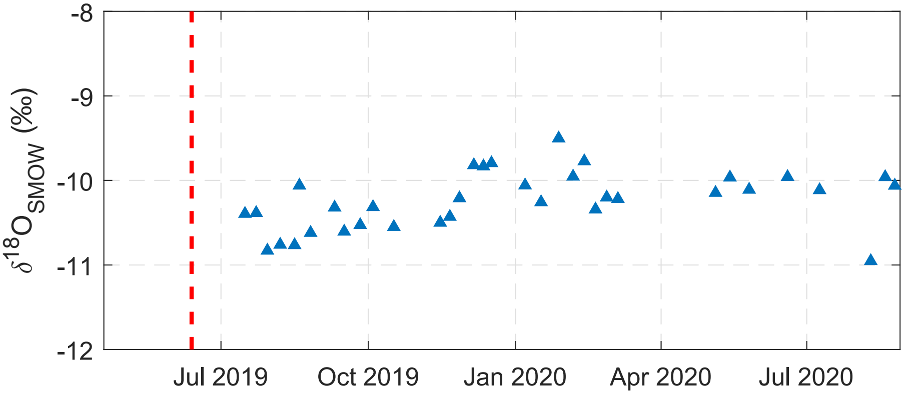

Fig. 1 δ^{18}O. Evolution of δ^{18}O during the experiment.

Full suit of ions

Fig. 2 shows the evolution of all ions measured.

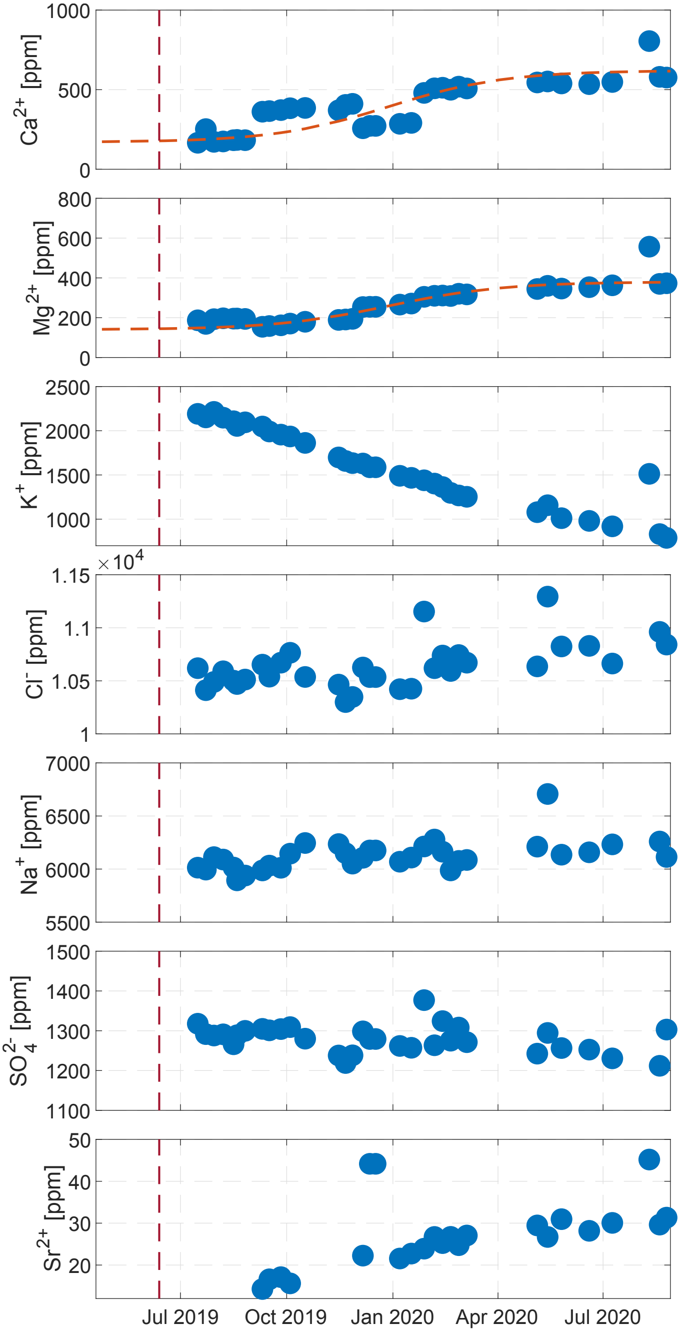

Fig. 2 Ion concentrations. Concentrations during the course of the experiment of [a] Ca, [b] Mg, [c] K, [d] Cl, [e] Na, [f] SO_4, [g] Sr. For Ca and Mg idealized lines are drawn in red. For Cl, Na and SO_4 the third last data point lies outside of the depicted range.
